# *“I’ll meet you at our bench”*: adaptation, innovation and resilience among VCSE organisations who supported marginalised and minoritised communities during the Covid-19 pandemic in Northern England – a qualitative focus group study

**DOI:** 10.1186/s12913-023-10435-5

**Published:** 2024-01-03

**Authors:** S Scott, VJ McGowan, J Wildman, E Bidmead, J Hartley, C Mathews, B James, C Sullivan, C Bambra, S Sowden

**Affiliations:** 1 Population Health Sciences Institute, Faculty of Medical Sciences, Newcastle University, Sir James Spence Building, Royal Victoria Infirmary, Newcastle upon Tyne, NE1 4LP UK; 2 ScotCen, Scotiabank House, 6 South Charlotte Street, Edinburgh, EH2 4AW UK; 3 Institute of Health, University of Cumbria, Fusehill Street, Carlisle, CA1 2HH UK; 4 VONNE, 4th Floor, MEA House, Ellison Place, Newcastle upon Tyne, Tyne and Wear, NE1 8XS UK; 5 Office for Health Improvement and Disparities, Department of Health and Social Care, Waterfront 4, Goldcrest Way, Newburn Riverside, NE15 8NY Newcastle upon Tyne, UK; 6 NIHR Applied Research Collaboration North East and North Cumbria, St Nicholas’ Hospital, Jubilee Road, Gosforth, Newcastle Upon Tyne, NE3 3XT UK

**Keywords:** Covid-19, Qualitative research, Voluntary sector, Public health, Health inequalities, Marginalised communities, Minoritised communities

## Abstract

**Background:**

The Covid-19 pandemic has exacerbated pre-existing inequalities and increased adversity and challenges for vulnerable and marginalised communities worldwide. In the UK, the Voluntary Community and Social Enterprise (VCSE) sector play a vital role in supporting the health and wellbeing of people who are marginalised or experiencing multiple complex needs. However, only a small number of studies have focused on the impact that Covid-19 had on the VCSE sector.

**Methods:**

As part of a Health Inequalities Impact Assessment (HIIA), we conducted qualitative focus groups with staff and volunteers from five organisations to examine short, medium and longer-term impacts of Covid-19 upon the VCSE sector in Northern England. Nine online focus groups were conducted between March and July 2021.

**Findings:**

Focus group transcripts were analysed using Framework Analysis and yielded three central themes: (1) exacerbation of pre-existing inequalities, adversity and challenges for vulnerable and marginalised populations; (2) the ‘price’ of being flexible, innovative and agile for VCSE staff and volunteers; and (3) the voluntary sector as a ‘lifeline’ - organisational pride and resilience.

**Conclusions:**

While the voluntary sector ‘adapted at pace’ to provide support during Covid-19 and in its continued aftermath, this resilience has potentially come at the cost of workforce and volunteer wellbeing, compounded by political obstacles and chronic shortage in funding and support. The VCSE sector has a vital role to play in the post-lockdown ‘levelling up’ agenda. The expertise, capacity and resilience of VCSE organisations, and their ability to respond to Covid-19, should be celebrated, recognised and supported adequately to maintain its resilience. To not do so threatens the sector’s sustainability and risks jeopardising attempts to involve the sector in addressing the social determinants of health.

**Supplementary Information:**

The online version contains supplementary material available at 10.1186/s12913-023-10435-5.

## Background

Since its emergence in late 2019, the Covid-19 pandemic has played out against a global backdrop of pre-existing social, economic, ethnic and geographic inequalities in existing non-communicable diseases (NCDs) and the wider determinants of health [1]. Numerous studies conducted in multiple countries have identified strong associations between Covid-19 mortality, socio-economic status, ethnicity, income inequalities and other axes of inequality and marginalisation [2–4]. As Bambra et al. [5] have argued, this is best described as a ‘syndemic’ relationship, where existing, co-occurring socio-economic inequalities interact with - and exacerbate - case rates, symptom severity, morbidity and mortality outcomes from the pandemic. This relationship has been compounded by inequalities, not just in terms of virus-related infection and mortality, but also in terms of the health consequences of pandemic policy responses undertaken in most countries, such as isolation, lockdown, economic and social policy changes, health care service restrictions and contact tracing [6, 7]. Thus, in the United Kingdom (UK), Covid-19 has impacted on regional areas in ways that mirror existing health and socio-economic inequalities [8], culminating in calls to ‘Build Back Fairer’ [9].

Whilst the origins and operations of Voluntary Community and Social Enterprise (VCSE) sectors differ from country to country, worldwide they play a vital role in supporting the health and wellbeing of people who are marginalised or experiencing multiple complex needs. Indeed, some have described them as ‘the last line of defence’ for those most vulnerable in our society [10, 11]. Whilst many studies have documented increases in the number of people supported and salience within specific countries, definitive global figures on the growth of VCSE sectors are not available as there is no single international repository of comprehensive statistics [12]. Our focus here is on the UK context, which has been described to have a significant and ‘vivid’ VCSE sector comparative to other countries [13, 14]. Over 168,000 charities appeared on the Charity Commission’s register in 2020, with a total income of £81.2 billion. As of 2021, the UK VCSE sector has a paid workforce of 951,611, and is disproportionately staffed by women and older adults; just under a fifth of all UK VCSE organisations work in social services. While substantially smaller than both the public and private sectors, the voluntary sector’s workforce has grown by 20% since 2010 - the fastest growth of any sector over the last decade. UK VCSE sector annual income currently sits at £56bn, half of which comes from the public, followed by more than a quarter from the government. It contributed approximately £20bn to the UK’s economy, or 0.9% of Gross Domestic Product (GDP). The social services subsector contributes the most, worth £3.8bn, with health worth £2.3bn [15].

The VCSE sector, often by working in partnership with other statutory or public agencies, have played a central role in responding to Covid-19 [16]. Those deemed to be minoritised or marginalised can become further marginalised during emergencies, in part due to the exacerbation of health conditions as a result of poor access to health services [17, 18]. Thus, Eshareturi et al. [19] found that the first UK lockdown led to a deterioration in the overall health of marginalised groups. Similarly, Stevens et al. [20] demonstrated that socially vulnerable groups faced particular financial hardship and destitution, and that digital exclusion, language and housing created barriers to following guidance. Further, Armstrong et al. [21] used multiple qualitative methods with four groups already experiencing exclusion, isolation and marginalisation in Scotland and found continuity of pre-existing hardship, as well as intensifying challenges through further constraint of already circumscribed lives. Meanwhile, Shakespeare et al. [22] have recently highlighted that the pandemic exposed and magnified existing inequalities and structural failings for disabled people, and that without VCSE organisations, things would have been far worse. Nevertheless, whilst there is an abundance of grey literature on this topic, and undoubtedly further academic research in the pipeline, to our knowledge, only a very small number of peer-reviewed published studies have focused on the impact of Covid-19 on the UK VCSE sector. Further, those that have, tended to report findings from earlier stages of the pandemic. Both Dayson et al. [23] and Cooney [24] have argued that Covid-19 has changed the context for many VCSE organisations who, particularly in earlier stages of pandemic restrictions, had to meet service delivery requirements (remaining socially connected with service users) while physically distancing. These challenges have been exacerbated as needs have changed or increased whilst, simultaneously, funding sources have been, at best, disrupted, with many providers forced to close or operate at reduced capacity during and beyond the pandemic lockdown periods [25, 26].

This paper therefore adds to an important emerging body of work and presents findings from one component (qualitative focus groups undertaken with VCSE organisations) of a ‘Health Inequalities Impact Assessment’ (HIIA) conducted in North East England. Thus, in part, some of the methods and findings included in this manuscript have been disseminated by collaborating partners previously as a short report [27] though not peer-reviewed or expanded upon in more detail as we do here. A HIIA is a systematic method of synthesising and gathering evidence to prompt consideration of the impacts of applying a proposed, new or revised policy or practice on differently placed people and the identification of actions required to ameliorate negative impacts [28]. It bears similarities to more traditional Health Impact Assessment (HIA) approaches, recommended by the World Health Organisation (WHO) as a practical approach used to judge the potential health effects of a policy, programme or project on a population, particularly on vulnerable or disadvantaged groups (https://www.who.int/tools/health-impact-assessments). We drew on the five key steps advocated by WHO within our own HIIA: screening, scoping, appraisal, reporting and monitoring. Sponsored by the North East Association of Directors of Public Health (NE ADPH), this HIIA was a collaboration between researchers from the NIHR North East and North Cumbria Applied Research Collaboration (NIHR NENC ARC), public health policy makers from the Office for Health Improvement and Disparities (OHID, formerly Public Health England [PHE]) and VCSE practitioners from the Voluntary Organisations’ Network North East (VONNE). Whilst the original geographical remit of this HIIA was North East England, the component of work reported here was broadened to include organisations in North Cumbria. The aims of the overarching HIIA were to: (1) understand the direct and non-direct health impact of Covid-19 on the population of North East England, including implications of the pandemic response for the most vulnerable and at-risk groups; and (2) assess this impact in order to inform health and social care recovery planning to support a focus on health inequalities and wider social determinants. Meanwhile, the aim of this particular component of work was to examine short, medium and longer-term impacts of Covid-19 upon the VCSE sector in North East England and North Cumbria.

## Materials and methods

Nine online focus groups with five VCSE organisations operational across the North East North Cumbria region were conducted between March and July 2021. Organisations were purposively sampled according to geographical location and one (of 4) pre-defined areas of vulnerability or risk developed by the North East Covid-19 HIIA steering group as part of their broader HIIA study. These four areas were: clinical complexity; increased risk of transmission; poor access to care and risk from indirect harm. Focus groups took place via a video conferencing platform (Microsoft Teams or Zoom) and were arranged by VONNE, who acted as a recruitment gatekeeper. Focus groups were moderated by SS, JW, VM and EB who worked in pairs. All moderators were, at the time of this study, female mid-career researchers (educated to PhD level), highly trained and experienced in qualitative data collection methods and analysis. To ensure that online discussion was manageable, focus groups were limited to a maximum of six participants. Participants could join using audio only or by using both video and audio. Focus groups were steered by a topic guide, guided initially by existing literature and themes identified in prior stages of the HIIA. Specifically, we explored: partnership models of working; short and medium-term adaptations and innovations as a result of the pandemic crisis (at strategic and ground level); organisational resilience, funding and sustainability; concerns about long-term future at sector and organisational level; and specific impacts of the pandemic upon staff, volunteers and key beneficiary groups. Nevertheless, this topic guide was iterative allowing space to continually re-evaluate emergent findings and perspectives. Several participants were known to the research team prior to this study; all participants in each focus group session knew each other. All participants received a study information leaflet, which included details about the researchers’ credentials and reasons for conducting this study; anonymity was assured. All focus group participants provided written informed consent to take part in the study. Ethical approval for this study was provided by the FMS Research Ethics Committee at Newcastle University (Reference: 8580/2020, 16th December 2020). To thank them for their time, VCSE organisations were paid £300 by PHE North East after participating in focus group sessions.

Eight (of nine) focus groups were audio-recorded and transcribed verbatim, with observational fieldnotes maintained in a research diary. One focus group was not recorded due to concerns raised over anonymity and safeguarding – but non-ascribed fieldnotes were taken. Primary data therefore comprised written material (focus group transcripts and fieldnotes). Focus group transcripts were analysed using Framework Analysis [29] and we followed the five stages outlined by Ritchie, Spencer and O’Connor [30], moving back and forth between stages throughout our analysis: (1) Familiarisation, (2) Identifying a thematic framework, (3) Indexing, (4) Charting, and (5) Mapping and interpretation. Independent coding and analysis were undertaken by SS, JW, VM and EB, with themes discussed and challenged at subsequent project meetings, using a process defined as pragmatic double coding [31]. Transcripts were first coded line-by-line and then systematically indexed into data tables to generate detailed descriptive themes. These descriptive themes were compared to identify patterns, similarities and differences in the data, and relationships between them elaborated, in order to generate analytical themes, and a consistent interpretation of the dataset as a whole.

Our approach to data collection, coding and analysis was guided by COnsolidated criteria for Reporting Qualitative (COREQ) research [32]. Our full COREQ checklist is included as Additional File 1. Several established approaches were also taken to ensure the validity and rigour of the findings including development of a coding system, peer review of themes, triangulation of multiple data sources and provision of thick description that recognises the context of data collection, supported by quotes and detailed field notes [33, 34].

## Findings

### Organisational and participant characteristics

In total, 39 adults (sampled from five VCSE organisations) took part, with focus groups comprising two to six participants per group. Nobody approached refused to take part or withdrew from the study. One organisation supported children and young people (CYP) (n = 8); two organisations supported vulnerable women, young people and families (VWF) (n = 16); one organisation supported refugees and asylum seekers (RA) (n = 10) and one organisation supported disadvantaged individuals to improve their mental and physical health and wellbeing (DMW) (n = 5). 82% (n = 32) of our sample were female, reflecting organisational remit and the needs of those supported by organisations who took part. Whilst we originally extended an invite to participate only to staff members, it became quickly clear that those volunteering within the organisation were embedded within staff groups. Thus, some focus group participants (n = 14) had prior lived experience of marginalisation and exclusion and had subsequently taken on a volunteer role at the organisation. Other focus group participants held different roles within organisations including project and/or team managers, key workers and advocacy/wellbeing/inclusion co-ordinators. Each focus group discussion lasted between 58 and 110 min. Analysis of focus group transcripts yielded three central themes: (1) exacerbation of pre-existing inequalities, adversity and challenges for vulnerable and marginalised populations; (2) the ‘price’ of being flexible, innovative and agile for VCSE staff and volunteers; and (3) the voluntary sector as a ‘lifeline’ - organisational pride and resilience. Sub-themes and themes are illustrated in a thematic map, presented as additional material (see Additional File 2, Figure S1).

The findings presented below include quotations to provide rich description and faithful accounts of the views and experiences of the participants in this study. All data were coded anonymously to ensure that participants were not identifiable from their accounts. Type of VCSE organisation is detailed alongside quotations to contextualise supporting extracts. We do not report further demographic information here in order to safeguard our participants and thus protect anonymity and confidentiality.

### Theme 1: exacerbation of pre-existing inequalities, adversity and challenges for vulnerable and marginalised populations

Exacerbation of inequality and adversary was imbued throughout all of our focus group narratives, which recognised the multiple, intersecting axes of inequality that those supported by the voluntary sector experienced during the pandemic including but not restricted to: unequal experiences of lockdown periods, unemployment, worsening mental health, and barriers accessing vital health and social care services. Thus, all staff and volunteers in our study expressed that Covid-19 had a predominantly detrimental impact on the populations that they supported, and that these impacts intersected in people’s lives, culminating in multiple social disadvantage and isolation. Specifically, they described the toll that pandemic restrictions, particularly long periods of isolation during lockdown periods, had on the mental health and wellbeing of disadvantaged communities.

*“For people who were able to continue to work like I was, I think there was some kind of normality we could hold on to, but there were people that had absolutely nothing, they had to be completely locked down, shut down, they had no work to go to and nothing*” (DMW VCSE organisation).

Further, VCSE staff and volunteers highlighted notable impacts in relation to access to services and support. For example, one contributor commented that beneficiaries were facing long waiting times when trying to call housing services, as well as a lack of awareness of where else to access help.

*“…when you call someone in the normal circumstances might be 10 minutes, five minutes, you have to wait, but during this time, the people have to wait 20 minutes, sometimes 40 minutes, so people, just like fed up and they are not trying again and they said no, no one is approaching us, we are struggling, but we don’t know where we need to go”* (RA VCSE organisation).

The move to digital services was also problematic for some beneficiaries. Whilst organisations supported beneficiaries to access services digitally, some found “general beginners’ level” too difficult, and others, for whom English was their second language, struggled to understand the tutors. Beneficiaries living rurally were reported to struggle with connectivity.

*“It’s access. A lot of them live in the countryside, so they just don’t have the Wi-Fi…they can get on but they just freeze, so it’s difficult.”* (CYP VCSE organisation).

Nevertheless, contributors also stressed that technology came with positives and brought benefits for staff, volunteers and beneficiaries. Staff highlighted differences between the first and second national lockdowns, but accounts tended to conflict on this point. Some suggested that the second lockdown period was received more positively as populations had come to terms with the pandemic and a vaccine was on its way. Others suggested the second lockdown was a ‘flat time’ for marginalised populations, who had lost hope. Either way, staff suggested that the legacy of Covid-19 was the exacerbation of pre-existing inequalities for the populations they support; this was not an equal pandemic. Some beneficiaries’ progress had halted or even reversed. Staff expressed sadness as well as anger and frustration, emotions which were replicated later when discussing the future of the sector.

*“At the beginning of the pandemic there was this expression that we’re all in the same boat and as the time went by that changed and people recognized suddenly that we’re in the same storm, but we’re not in the same boat, some people have yacht, some people have got in different vessels to weather the storm”* (RA VCSE organisation).

### Theme 2: the ‘price’ of being flexible, innovative and agile for VCSE staff and volunteers

Like the communities they supported, VCSE staff and volunteers were impacted by the pandemic in multi-faceted ways, both professionally and personally. Correspondingly, they were required to flex and adapt in order to continue to support those that needed it. Therefore, the Covid-19 pandemic represented both a challenge and a learning curve (“*a bit of a rollercoaster*”), and has presented positives and disadvantages for the sector, staff and volunteers. It has been an intense period of time, leading to increased workloads (and the complexities of furlough), anxiety and stress, and culminated in exhaustion from juggling competing responsibilities of work and family life. Whilst all focus group participants in our study described pandemic impacts for themselves and populations they support, this was more pronounced in the narratives of participants with lived experience, most prominently those supporting vulnerable women, young people and families (VWF); and refugees and asylum seekers (RA).

*“I was working myself to exhaustion… it took me to catch the coronavirus and when I was done, I realised that you know, if you don’t look after yourself nobody will. So, I learned self-love in a hard place.”* (RA VCSE organisation).

Nevertheless, there were unexpected positives and surprises, such as the encouraging reactions from funders, the widespread human kindness that was apparent and having time to reflect and spend time with family. Deeper connections with beneficiaries and colleagues were also reported. Both staff and volunteers articulated feeling grateful and more mindful as a result of lockdown. One contributor believed the open discussions about mental health that took place during the pandemic would bring societal benefits from greater acceptance of poor mental health.

*“It’s not a positive thing that young people are experiencing these feelings but it’s a positive thing for society… there’s a lot of people who still don’t believe mental health problems are real, for them to see that in maybes their children or themselves then that could be a positive thing in in the longer term. For a bit more of acceptance in society…”* (CYP VCSE organisation).

VCSE staff and volunteers stressed that the Covid-19 pandemic meant that, as a sector, they had to be agile, adaptable and innovative in service delivery, particularly during the early stages of lockdown, which happened very fast and came with a great deal of anxiety. Many stressed that it was particularly important during this time to meet the diverse needs of their beneficiaries. Here, staff reflected on the lengths they went to in order to ensure that everyone (staff, volunteers and beneficiaries) had access to technology and access to support, both of which were framed by social distancing restrictions. As a sector, this resulted in a great deal of organisational learning. Moreover, VCSE staff were said to have performed “magnificently”, to have gone “above and beyond” what would have normally been expected in order to keep delivering support to beneficiaries. The new, flexible ways of working devised made some contributors reflect that perhaps their previous ways of working had not been as accessible as they thought. One contributor talked at length about the park bench that had become a new regular meeting place with an established client (“*You know you just wouldn’t have had this 18 months ago, it will be like I see [you] two o’clock at the office but he’s saying to me “I’ll meet you at our bench”)*, whilst others discussed the necessity for walking meetings and home visits rather than office-based appointments.

*“We are offering walk and talk appointments out in the community… we would meet outside the office and go for a walk along the river for an hour or so, or in the park or even through the middle of [name of area] when the coffee shops were open and grab a coffee halfway, just having that bit of human contact. One of the clients put off doing one for quite some time, and when she did one she enjoyed it. One of the client’s feedback was that she felt quite normal. Yes, I think we’ve been quite good at adapting how we can work across the board.”* (VWF VCSE organisation supporting vulnerable women).

Nevertheless, reductions in face-to-face contact and/or working remotely resulted in impacts and challenges such as reduced engagement and uptake from both new and existing people within their services, alongside higher referrals and need. Staff reported concern relating to the potential exacerbation of harm for some marginalised populations such as sex workers and those experiencing domestic abuse. A lack of IT support and digital exclusion was also reported.

*“I think the biggest struggle for me was the lack of IT support for participants. Because everything was directed online… and not a lot of people who are on benefits have the facilities to do that … so we’ll provide you with a tablet or the government will give you this, you can even get this broadband for really, really cheap money. Right okay, so I don’t have a tablet. I don’t have a smartphone to access this broadband that you’re going to give me for a really good price and then it’s finding some way to support that … we managed to get some tablets and smartphones with data and stuff and that helped for a little bit… even now it’s all still online and it’s a big problem for a lot of people getting online and learning…”* (DMW VCSE organisation).

On a more personal level, there was a tension between staff and volunteers who had relished the flexibility of working remotely and/or at home compared to some that had found doing so incredibly difficult. Indeed, many were keen for such a high degree of flexibility to be maintained long-term, post-lockdown; others could not wait to get back in the office. Home working came not just with technology issues, but also with issues framed around being able to ensure confidentiality.

*“I didn’t enjoy working from home… I found it very difficult because I live with my husband and my son and his family, the children were home… I just felt very uncomfortable talking about the confidentiality of cases… if you’ve got the police on the phone and suddenly you’ve got family walking in… I felt like I was 22 invading my grandchildren’s space… when second lockdown happened, I was asked, and my colleague was asked if we want to work from home, and I said no. I don’t want to work from home because I feel I cannot work with a woman, in terms of giving full confidentiality and capacity*” (VWF VCSE organisation).

Further, moving between remote and face-to-face delivery came with its own set of challenges such as bubble systems, limits on group numbers, mask wearing, social distancing and engagement. This was predominantly articulated by those responsible for youth provision, who historically thrived in working with large groups.

*“… It’s actually easier to be working outside right now… we don’t wanna turn young people away but we’re having to… young people are like, ‘Well we don’t have to do this at school, we don’t have to wear a mask at school… I go to school with them, so I don’t have to stay away with them, I’m in a bubble’, and I’m like ‘Yeah, but you’re not in a bubble here’…”* (CYP VCSE organisation).

### Theme 3: the voluntary sector as a ‘lifeline’ - organisational pride and resilience

VCSE staff and volunteers articulated uncertainty about the future, particularly in relation to funding. Specifically, they felt that the centralisation of funding for the Covid-19 response had interrupted funding for providers’ everyday business. Nevertheless, staff and volunteers discussed the advantages gained from working in partnership with other organisations; and technology had facilitated the establishment and nurturing of networks and partnerships. However, new organisations and groups had also developed to help with the Covid-19 response, and this had increased competition; contributors believed this could have long-lasting repercussions for the sector in terms of financial planning at a time when there was increasing need, pressure and a high number of referrals. Staff and volunteers also believed that the pandemic had exposed and exacerbated existing tensions between the voluntary and statutory sectors; and, in particular, one contributor referred to the NHS developing interventions without collaboration with the third sector.

“*I think that, you know, the whole thing around how, especially the community level, organisations have come together and pooled resources to deliver. I’d like to see more of that happening, but I think again, that could be driven by funders and by commissioners, that they actually, you know, were more proactive about commissioning and funding them types of models of working…”* (CYP VCSE organisation).

“*There’s a lot of work trying to be done around coordination and partnerships, but I think there’s always going to be too many people applying for funding.”* (VWF VCSE organisation).

*“… hopefully, in the future they’ll realize that they do need to speak to people before they make decisions and push ahead with ideas that they think are right… I know that isn’t what the community needs… it’s very, very frustrating. But this is just one of many examples where that’s happened…”* (DMW VCSE organisation).

There was widespread sector and organisational level pride apparent; and peer-to-peer support from colleagues was especially recognised. Staff and volunteers recognised how important they were in the lives of the communities they supported. Nevertheless, contributors were tired and there appeared to be some tensions with regards to working so hard whilst other colleagues were furloughed.

*“… we’ve bloody kept fighting…It’s literally just dragging yourself through each week… as a team we’ve been really supportive of each other, not just professionally but personally as well which I think has really helped.”* (CYP VCSE organisation).

In line with the previous theme on the need to be flexible and agile, whilst staff and volunteers felt they had enough support, they expressed that they would have liked greater appreciation for their efforts over the past 18 months, both internally and externally.

*“For the voluntary sector, I think it’s just another example of how quickly we can adapt. I know that you can’t really base your funding on that, but I do think we’ve got so much flexibility and different ways of engaging with some of those people who haven’t yet engaged in services. I sometimes feel like the voluntary sector is seen as, we’re just a bunch of do gooders and we don’t have the policies and we don’t have the procedures in place, but you know we do. And even though we still abide by all of those we can be really, really flexible in our approaches and I think the last year has evidenced that more and more”* (VWF VCSE organisation).

## Discussion

Our research found further evidence that the pandemic exacerbated pre-existing inequalities, and increased adversity and challenges for vulnerable and marginalised communities. This was especially the case in relation to the toll of restrictions on mental health and wellbeing; barriers to accessing services and support; and challenges related to digital exclusion. These findings reinforce a now vast body of research that has highlighted the collateral health damage of policy responses to the pandemic [6]. Nevertheless, our focus in this work was VCSE staff and volunteers, who have played a central role in supporting already marginalised communities during the Covid-19 pandemic [16]. Perhaps most importantly, our findings highlight that the adversity and challenges faced by beneficiaries were mirrored in some ways amongst the staff and volunteers who supported them, with the need to continually flex and adapt in order to offer enough support taking its toll on workers, who experienced an array of professional and personal impacts. This is a path less trodden in research – we know much less about the repercussions of the pandemic for VCSE staff and volunteers, particularly at a time of crisis. Recent focus groups undertaken with the penal voluntary sector have also illustrated the emotional labour undertaken by VCSE practitioners in order to mitigate experiences of anger, frustration, sadness and disappointment [11]. Meanwhile, research focusing on healthcare staff has recognised the cost of caring at times of crisis, including during the pandemic [35, 36]. We also noted that impacts were more pronounced in the narratives of participants with lived experience, most prominently those supporting vulnerable women, young people and families (VWF); and refugees and asylum seekers (RA). Whilst terminology can vary, here we draw on Buck et al. [37], who define ‘lived experience’ as direct personal experience of a social issue/issues. Other studies have highlighted both the impact and importance of lived experience within the VCSE sector [38, 39]. Whilst we are within broad agreement with this, our findings illustrate the potential for experiences, impacts and challenges to re-trigger or exacerbate existing hardship, emotional scars or trauma, a field of study which warrants further attention post-lockdown [37, 40, 41].

### Implications for future research, policy and practice

Our study sheds further light on the radically changed context in which the VCSE is operating [23]. VCSE staff and volunteers in our study had to be adaptable and innovative in finding new ways to deliver services. Indeed, the ability to rapidly adapt to unprecedented disruption was a source of pride and an important facet of the widely reported organisational resilience, despite the tolls outlined above. Further, the requirement to adapt brought new opportunities. Many organisations had found innovative ways to reach clients and reported that some practices were likely to be retained post-lockdown, including walking meetings and increased use of online services. Whilst the widespread shift to remote service provision necessitated by the pandemic provided new opportunities, our focus group participants identified that this also created challenges for beneficiaries without Internet access. The move to ‘digital first’ services (for example, the proposed direction for England’s primary care provision (see NHS England » Digital First Primary Care), risks further marginalising vulnerable people who, for whatever reason, lack the capacity to access the Internet [18, 42, 43]. Therefore, it is vital that rigorous inequalities impact assessments are conducted before any services are moved to online only. Meanwhile, achieving organisational resilience through staff effort and ingenuity can only ever be part of the picture; VCSE organisations also require adequate and dependable funding streams. Funding constraints in the sector inevitably reflect wider socioeconomic inequalities discussed earlier in this manuscript and which form the context to this work. Put simply, it is not a level playing field for marginalised communities nor for the organisations that support them. Yet, charities suffered a £6.6 billion reduction in funding during the pandemic [44]. Meanwhile, research with VCSE leaders stressed the importance of recognising the link between the wellbeing of staff in the sector and the impact that their work can have, with a call for funders to recognise this in their funding [45]. Our study participants identified threats to funding from increased competition for ever more scarce resources combined with a short-term focus on specific pandemic-related needs at the expense of beneficiaries’ existing and on-going needs. Indeed, the precarious position of the VCSE has long been recognised, with Villadsen [46] highlighting their almost ‘mythical’ reputation as ‘problem solvers’, who are oft championed for an ability to fill the cracks that public services cannot reach. However, this is not sustainable on a long-term basis and is subject to rupture as the pandemic has demonstrated. As Dagdeviren et al. [10] argue, such political obstacles and chronic shortage will radically reduce the sector’s ability to support vulnerable people and communities, and a resilient VCSE sector will require dependable, long-term and unrestricted funding that can be used to address local needs [47, 48].

Finally, the ability of the VCSE sector to respond at speed and with imagination to the Covid-19 crisis demonstrates the importance of involving the sector in pandemic recovery planning [49, 50]. Participants in our study expressed frustration that their intimate knowledge of their local populations’ needs was often not acknowledged by statutory sector organisations. To this end, Bynner et al. [49] argue that the relational skills of the voluntary sector are needed to supplement local statutory services to provide a sustainable response to the needs of vulnerable and marginalised populations. There is also a need for a strategic and complementary relationship between the state and voluntary sector, one that fully engages locally embedded voluntary organisations at all stages of emergency response and resilience planning [49]. Although the vital role of the VCSE in the pandemic response has been recognised [51, 52], it has not been accompanied by funding. A similar critique has been levelled at recent attempts to engage the VCSE sector in playing a role in improving health through ‘social prescribing’ interventions, which link patients with non-clinical VCSE resources to improve lifestyle behaviours. While the VCSE provide many of the services to which patients are linked, they rarely receive additional funding to provide these services [53]. The failure to properly recompense the VCSE sector for its services threatens the sector’s sustainability and risks jeopardising attempts to involve the sector in addressing the social determinants of health [54]. If the VCSE sector is to play a meaningful part in supporting continued Covid-19 recovery, then a seat at the table must be accompanied by funding. In practice, this means the VCSE sector should be a partner in strategic planning, should be funded adequately to deliver services and the VCSE workforce should be valued for the expertise that they bring. To this end, we align with Shakespeare et al. [22], who contend that the precarity of the VCSE sector must be addressed in three key ways in order to ensure it can continue to provide support: by working with the sector as equal partners rather than contractors; by reducing unnecessary reporting and administration; and by providing fair and longer-term funding.

### Strengths and limitations

This study is one of only a small number of studies which have focused on the impact of Covid-19 upon the VCSE sector. Our findings also advance the field methodologically in two distinct ways. First, our work represents a collaborative approach to the design and conduct of research and consolidated a developing partnership between researchers, public health policy makers and VCSE practitioners. This allowed for a co-produced and egalitarian mode of data generation with values and principles for greater equality embedded, a strength now widely recognised in existing literature [55]. Findings from qualitative focus groups with VCSE organisations reported here formed an integral component to a wider HIIA process and directly informed policy and practice recommendations in North East England. Thus, by adopting a co-produced approach to the design and conduct of a HIIA, we have been able to obtain action-orientated insights into the perceived problems and solutions for Covid-19 recovery planning from the perspective of VCSE staff and volunteers.

Second, conducting a HIIA remotely demonstrates a potentially novel approach to the conduct of a HIIA, and one which could be adopted in future impact assessments. Qualitative studies have shown remote platforms such as Microsoft Teams and Zoom to be effective in conducting interviews due to the user friendliness, convenience and ease of creating rapport through the screen [56, 57]. A limitation of this study is that all recruitment took place via a VCSE sector regional support body that represents organisations in North East England. This recruitment strategy may have failed to reach grassroots organisations that do not have connections with formal umbrella organisations. Further, our study may not reflect the experiences of VCSE in the other regions of England or internationally. Secondly, focus group participants may have felt apprehensive discussing their views in front of others and there may have been things that people did not say. In this study, focus group participants were part of the same organisation and were not mixed across different organisations. At the beginning of each focus group we stated there were no right or wrong answers; that participants should respect people with different perspectives and that the focus group was a safe space to express views. Further, we also attempted to engage with people using the chat function which may have offered people the opportunity to express their views in a less exposing way. Whilst we recognise that such processes may still not have been sufficient to allow people to feel comfortable to talk freely in front of others, as we collected data from one organisation at a time, we feel confident that this aided open discussion, particularly as participants already knew each other. Further, whilst we focused on medium to longer term pandemic impacts, our study was conducted whilst lockdown restrictions remained operational in England. Now that restrictions have been fully lifted, day-to-day working environments for VCSE staff and volunteers will be inevitably altered. Finally, within our study, we did not explore what the situation could have been like for marginalised and minoritised communities without lockdown restrictions. Whilst data does exist on countries that didn’t lock down or which locked down later, country context affects Covid outcomes making it difficult to make comparisons. Further, we found no extant studies which differentiated between locking down and not locking down in the UK and explored the impact this might have had specifically upon inequalities experienced by those supported by the types of organisations taking part in our research. Studies that do exist, however, acknowledge that the pandemic (and lockdown measures) did exacerbate existing health and social care inequalities in England across numerous markers. Nevertheless, modelling studies using general population UK data do suggest that lockdown scenarios are the most stringent way to slow transmission (and thus reduce hospital admissions, disability and deaths) [58–60]. Meanwhile, Arnold et al. (2022) specifically modelled the importance of how lockdown measures were timed, suggesting that introducing measures one week earlier would have reduced by 74% the number of confirmed Covid-19 cases in England by 1 June, resulting in approximately 21,000 fewer hospital deaths and 34,000 fewer total deaths; the required time spent in full lockdown could also have been halved, from 69 to 35 days.

## Conclusion

As we move into a world where organisations must ‘live with Covid-19’, prolonged impacts of the pandemic on the VCSE sector and its beneficiaries are not yet fully understood. There remain significant knowledge gaps and ‘unknowns’ still to tackle, and we are yet to find out the real, long-term impacts of the pandemic on marginalised communities. However, the VCSE sector has a vital role to play in the post-lockdown ‘levelling up’ agenda [48]. The relational expertise, capacity and resilience of VCSE organisations, and their ability to respond to crisis, including Covid-19, should be celebrated. This has never been more important than in the current UK context, a time of health and wellbeing crises in multiple directions - increasing inequalities, increasing poverty and socio-economic inequality, cost of living concerns, and a growing climate emergency - all of which were already growing in intensity before the pandemic hit. This is likely to include responding to longer-term health and social care impacts of Covid-19, including ‘Long Covid’, the impacts of which we still know very little about, but which will arguably lead to an even greater need for additional VCSE support and funding. Statutory and funding bodies must therefore ensure that the VCSE sector (including its staff and volunteers) are supported adequately to maintain its resilience and its ability to support communities that need them the most.

### Electronic supplementary material

Below is the link to the electronic supplementary material.

Supplementary Material 1

Supplementary Material 2

## Data Availability

The datasets used and/or analysed during the current study are available from the corresponding author on reasonable request.

## List of abbreviations

COREQ: COnsolidated criteria for Reporting Qualitative Research
CYP: Children and young people
DMW: Disadvantaged individuals, mental, physical health and wellbeing (DMW)
GDP: Gross Domestic Product
HIA: Health Impact Assessment
HIIA: Health Inequalities Impact Assessment
NCDs: Non-communicable diseases
NE ADPH: North East Association of Directors of Public Health
NIHR NENC ARC: NIHR North East and North Cumbria Applied Research Collaboration
OHID: Office for Health Improvement and Disparities
PHE: Public Health England
RA: Refugees and Asylum Seekers
UK: United Kingdom
VCSE: Voluntary Community and Social Enterprise
VONNE: Voluntary Organisations’ Network North East
VWF: Vulnerable women, young people and families
WHO: World Health Organisation
