# Supplementary material for: “I’ll meet you at our bench”: adaptation, innovation and resilience among VCSE organisations who supported marginalised and minoritised communities during the Covid-19 pandemic in Northern England – a qualitative focus group study
Source: BMC Health Serv Res. 2024 Jan 3;24:7. doi: 10.1186/s12913-023-10435-5 (PMC10765907; doi:10.1186/s12913-023-10435-5)
Supplement: Supplementary file 2 — Supplementary Material 2 [file 12913_2023_10435_MOESM2_ESM.docx]

**Figure S1: Thematic map illustrating relationships between themes and sub-themes**
